# Notes and News

**Published:** 1894-04-21

**Authors:** 


### Notes and News.

Collected and Communicated.

MEETINGS OF THE WEEK.

Lock Hospital, Hanover Road, Festival Dinner, Hôtel Metropole, Saturday, April 21st.
Princess Louise Home, Kingston Hill, Surrey, Distribution of Prizes, Saturday, April 21st.

The thirty-fourth annual dinner of old students of King's College, London, will take place on May 30th at the Holborn Restaurant, when the chair will be taken by Sir Richard Webster, Q.C., M.P.

In France cremation is gaining a decided hold. At Père Lachaise it is so much resorted to that it is proposed that all the Paris burying grounds should be equipped for the adoption of this means of disposing of the dead.

Worthing has, indeed, paid dearly for its neglected sanitation. Apart from the general depression it has caused to all inhabitants more or less, the amount of money expended directly and indirectly has been enormous. The Corporation, now really roused to the necessities of the case, are spending £50,000 in drainage works and £20,000 in new waterworks, They have rejected M. Hermite's system of electric sanitation.

Although in England the highest honours are not reserved for those who distinguish themselves in medicine, on the Continent recognition is more liberally awarded. Dr. Sinclair Coghill, the senior physician to the Ventnor Hospital for Consumption, has been awarded the diploma and insignia of the Fourth Class, Knight Commander of the Royal Servian Order of St. Sava by King Alexander of Servia. The order is given for distinction in science, literature, and affairs.

In the large parish of Islington the vaccination laws appear to be a dead letter. The same seems to be the case in Mile End. The vice-chairman of the Mile End Board of Guardians is reported to have declared the other day that defaulters in the district number altogether 6,500. And what, meanwhile, about the Vaccination Commission? It has been sitting for six years, a contemporary points out, and the final report has still to come.

The healthiest town in England, for the moment, is Croydon, which has a mortality of only 11·2 per 1,000 per annum. In Brighton, for the week ending February 21st, there was a death-rate of 15·8; and in London, 18·6.

It would seem that Glasgow, in spite of initial opposition, is destined to be one of the chief centres of encouragement to medical work amongst women. Five thousand pounds has now been granted by the trustees of the Bellahouston estate towards the erection and fitting of buildings for medical and scientific instruction at Queen Margaret's College.

Although evidence before the Opium Commission has been closed for some time, memorials are still being sent in from those interested. For instance, the members of the Burmah Branch of the British Medical Association, have memorialised their opinion that further restrictions in the sale of opium are not only needless but would be harmful, and that in Burmah the regulations are sufficiently stringent, and that further prohibition would lead to the consumption of more harmful substitutes, such as alcoholic stimulants and hemp drugs.

The laws in Switzerland appertaining to inebriates are going through various phases of legislative evolution. These contain the following clauses: Art. xxv.—If the crime is attributable to the use of spirituous liquors, the judge shall have power to prohibit the admission of the accused to public-houses for a period of from one to five years. Art. xxvi.—If the permission of the drunkard to a special establishment is indicated the judge orders it on medical advice, independently of a period of detention varying from five to six years.

The sum of £16,000 is still required to pay for the new buildings of the Great Northern Central Hospital, which are shortly to be opened. It is hoped that £5,000 will be the direct result of the bazaar which is being held this week in the new wards. The bazaar was opened on Wednesday by H.R.H. the Duchess of Teck, who afterwards presided at one of the stalls. H.R.H. the Duchess of Albany was present on Thursday, and on Friday her Grace the Duchess of Devonshire kindly consented to open the bazaar.

It has been proposed to establish a Society of Fellows of the College of Surgeons. A meeting has been held to consider the matter. Mr. Timothy Holmes, Mr. Warrington Haward, Mr. Treves, Mr. Rushton Parker, and others spoke. Mr. H. Page occupied the chair, and explained the reasons for proposing the scheme. The strongest motive seemed to be the regeneration of interest on the part of the Fellows in the College by establishing the society. Over 245 Fellows seemed favourable to the idea, in response to a circular on the subject. Mr. Timothy Holmes spoke at some length against the movement. He pointed out that the Fellows had the right of meeting by themselves, and that unless they disapproved of the proceedings of the College they would not be benefiting it or themselves by forming a separate society amongst themselves. The motion was carried with six dissentients, and a resolution to form a central executive council in London, empowered to enter negotiations with Fellows of the College in various centres for the formation of branch executives, was also passed. Messrs. F. Treves, H. Page, C. H. Golding-Bird, W. H. Bennett, Anthony Bowlby, Bernard Pitts, George Eastes, John Morgan, and F. Wallis were elected on the executive council.

